# Inhibition of *GATA4* and *MEF2C* Induced by Hypo‐Acetylation of Histone H3 Partly Reverse Heart Failure in Mice

**DOI:** 10.1002/pdi3.70045

**Published:** 2026-03-27

**Authors:** Xinru Gan, Bo Pan, Lingjuan Liu, Mi Li, Huichao Sun

**Affiliations:** ^1^ Department of Cardiovascular Medicine, Ministry of Education Key Laboratory of Child Development and Disorders, National Clinical Key Cardiovascular Specialty, Chongqing Key Laboratory of Structural Birth Defect and Reconstruction, Key Laboratory of Children's Important Organ Development and Diseases of Chongqing Municipal Health Commission, National Clinical Research Center for Child Health and Disorders Children's Hospital of Chongqing Medical University Chongqing China

**Keywords:** *GATA4*, heart failure, histone acetylation, *MEF2C*

## Abstract

The pathogenesis of heart failure involves a highly intricate process regulated by diverse epigenetic factors, transcription factors, noncoding RNAs, and cyclins. Notably, the reexpression of embryonic cardiac transcription factors, including *GATA*, *MEF2*, and *Nkx2.5*, is considered to exert critical influence in the initiation and advancement of heart failure. Nevertheless, the precise mechanisms through which epigenetic modifications drive this reprogramming of gene expression remain poorly defined. This investigation aims to clarify the role of histone acetylation in regulating the reexpression of embryonic cardiac transcription factors during heart failure. Our research indicates that during heart failure of mice, there are distinct histone acetylation modifications associated with the reexpression of these factors. Notably, *GATA4* and *MEF2C* show significant increases, whereas *Nkx2.5* shows a decrease compared to normal groups during heart failure progression. These findings imply that embryonic *GATA4* and *MEF2C* may promote the development of heart failure, whereas *Nkx2.5* does not appear to participate in disease progression. Furthermore, treatment with curcumin, a known inhibitor of histone acetylation, reduces acetylation levels at H3K4, H3K9, and H3K27 within the promoter regions of *GATA4* and *MEF2* *C* in a murine model of heart failure, leading to downregulation of these genes and subsequent enhancement of cardiac performance. In summary, our study demonstrates that p300 exerts site‐specific regulatory effects on various transcription factors via histone modifications, and low acetylation status at specific sites can inhibit reactivation of *GATA4* and *MEF2C* during the myocardial dysfunction period thereby improving cardiac performance of mice.

## Graphical abstract

Embryonic *GATA4* and *MEF2C* may contribute to the development of heart failure while Nkx2.5 may not be involved in the progression. Treatment with the histone acetylation inhibitor curcumin can reduce the expression levels of *GATA4* and *MEF2C* by inhibiting acetylation at H3K4, H3K9, and H3K27 sites, leading to an overall improvement for heart failure symptoms.

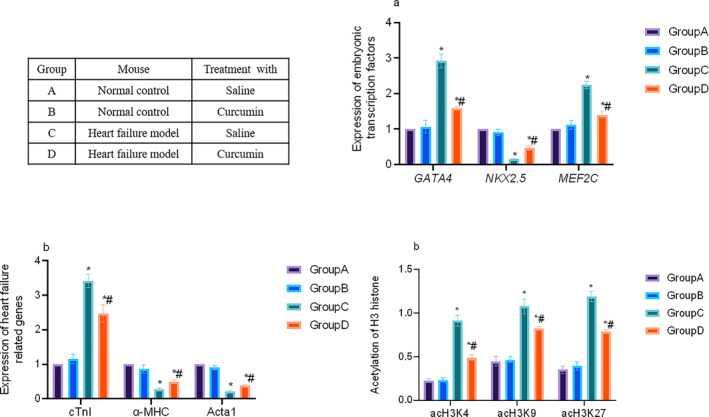

## 1 Introduction

Heart failure (HF) is a clinical syndrome marked by structural and functional impairments of the heart, arising from diverse etiologies such as diabetes, hypertension, myocardial infarction, ischemic cardiomyopathy, obesity, smoking, and genetic predisposition [1]. These abnormalities compromise the heart's pumping capacity, leading to inadequate perfusion to meet the metabolic demands of peripheral organs and tissues. HF imposes a substantial burden on global health and the economy. Although medical science has always been committed to research on HF, this condition remains one of the most rapid cardiovascular diseases in the world, with an associated mortality rate approaching 50% [2, 3, 4, 5, 6]. In adult mammals, the heart's capacity for regeneration and repair is severely limited, which represents a major obstacle to restoring cardiac structure and function after injury, thereby driving the progression and worsening of HF [7]. At present, there is no cure for HF apart from heart transplantation. However, prohibitive costs and scarce donor availability restrict access for many patients, underscoring the urgent need for novel therapeutic strategies [8].

Studies have found that HF is accompanied by a marked reactivation of “fetal” gene programs and suppression of “adult” genes. These phenomena are linked to compensatory cardiac remodeling, though the underlying mechanisms remain poorly defined [9]. “Fetal type” genes include, amongst others, *GATA4*, *MEF2C*, and *Nkx2.5*, which are highly expressed and functionally pivotal during embryonic heart development and then decline after birth and stabilize at low levels. Alterations in the expression or mutation of these genes not only contribute to cardiac malformations and congenital heart disease but also play a significant role in the pathogenesis and progression of HF in adulthood [10, 11, 12]. Shimizu et al. found that *GATA4* was overexpressed in the hearts of mice by transgenic technology, and myocardial hypertrophy was observed as a result, indicating that postnatal reactivation of *GATA4* may promote myocardial remodeling and participate in HF pathophysiology [12]. Transgenic mice overexpressing *MEF2C* developed ventricular dilation and contractile dysfunction. In contrast, mice expressing a dominant‐negative variant of *MEF2C* have shown delayed onset of HF, myocardial hypertrophy, and ventricular dilation under identical stress conditions [13]. These results indicate that reactivation of *MEF2C* is linked to HF pathogenesis, and its inhibition may attenuate disease progression. Similarly, transgenic adult mice overexpressing *Nkx2.5* have exhibited upregulation of ANF, BNP, and Myl2, suggesting a potential role for *Nkx2.5* in HF [14, 15]. Collectively, these findings suggest that postnatal reexpression of fetal genes including *GATA4*, *MEF2C*, and *Nkx2.5* may contribute to myocardial remodeling and HF. However, the specific mechanisms remain unclear, for instance, how are these “fetal” genes reactivated and how do they regulate the gene expression downstream to cause myocardial remodeling, ultimately leading to heart failure. These are all important questions in the field of cardiac research.

Epigenetic mechanisms, particularly histone acetylation, significantly influence gene expression patterns in HF [15]. The histone acetyltransferase p300, integral to cardiac development, catalyzes lysine acetylation on histone tails, promoting chromatin relaxation and transcriptional activation. Inhibition of p300 has shown to downregulate fetal gene expression and cause fetal cardiac maldevelopment [16, 17]. Conversely, p300 overexpression is associated with cardiac hypertrophy and HF [16, 17, 18, 19]. Studies demonstrate that p300 facilitates *GATA4* polymerization, activating transcription of hypertrophy‐related genes and promoting cardiomyocyte hypertrophy and HF [16, 17, 18, 19]. Moreover, p300‐mediated acetylation at H3K4, H3K9, and H3K27 has been implicated in modulating the expression of *GATA4* in cardiomyocytes [20]. However, the involvement of histone acetylation in the reactivation of fetal genes during HF, along with its precise regulatory mechanism, remains unclear. The role and mechanisms of histone acetylation in the reprogramming of fetal gene expression during HF were probed in our research. By revealing these mechanisms, we hope to identify potential therapeutic targets for reversing or preventing maladaptive processes associated with HF.

## 2 Materials and Methods

### 2.1 Animals

Male C57BL/6 mice, aged 6 to 8 weeks (20–25 g, specific pathogen free [SPF] grade), were sourced from the Experimental Animal Center of Chongqing Medical University (Chongqing, China). All experimental protocols involving animals were reviewed and approved by the Animal Care and Use Committee of Children's Hospital of Chongqing Medical University (Approval No. CHCMU‐IACUC20220629001) and conducted in compliance with the Guide for the Care and Use of Laboratory Animals.

### 2.2 HF Model

Heart failure was induced via intraperitoneal injection of isoprenaline [21], which was sourced from Sigma‐Aldrich (Missouri, St. Louise, USA). The mice were randomly assigned to two groups: a normal control group (*n* = 10) and an HF model group (*n* = 10). One of normal control group was given intraperitoneal injection of physiological saline (*n* = 5, Group A); another control group was given intraperitoneal injection of curcumin (*n* = 5, Group B). Mice in the HF model group were further divided into two groups: intraperitoneal injected with saline (*n* = 5, Group C) and intraperitoneal injected with curcumin, 70 mg/kg/d (*n* = 5, Group D). The intraperitoneal injection was continued for 28 days.

### 2.3 Treatment of Mice With HF

Mice in the HF group were further subdivided into two groups: Group C (intraperitoneal injected with saline, *n* = 5) and Group D (intraperitoneal injected with curcumin, 70 mg/kg/d, *n* = 5). The intraperitoneal injection was continued for 28 days. Body weight (BW), tail length, and heart weight (HW) were recorded, after which blood and cardiac tissue samples were collected for further analysis.

Serum concentrations of cTnI, CK‐MB, and NT‐proBNP were quantified using commercial ELISA kits (Shanghai Jianglai Biotechnology, Shanghai, China) to confirm HF induction. Blood samples were collected in serum separation tubes, incubated at room temperature for 2 h or at 4°C overnight, and subsequently centrifuged at 1000 g for 20 min. The supernatant was collected for subsequent assays. The experiment process was carried out according to the kit protocols.

### 2.4 Histological Analysis

Cardiac tissues were harvested at the end of the experiment, rinsed, and fixed in 4% paraformaldehyde. Following dehydration, clearing, and paraffin embedding, sections were cut at 5 μm thickness. Tissue slices were deparaffinized, rehydrated, and stained with hematoxylin and eosin (H&E) in line with conventional procedures. Imaging was performed using a Leica TCS SL microscope Version 2.0 (Wetzlar, Hessen, Germany).

### 2.5 Western Blot

Nuclear proteins were isolated from cardiac tissue samples employing a Nuclear Extract Kit (Thermo Fisher Scientific Inc, Waltham, MA, USA) and quantified with a BCA protein assay kit (Thermo Fisher Scientific, Waltham, MA, USA). Western blotting was performed using antibodies against acetyl‐histone H3 (Lys4), acetyl‐histone H3 (Lys9), acetyl‐histone H3 (Lys27), and total histone H3 (all from Abcam, Waltham, MA, USA). Signals were visualized with an enhanced chemiluminescence luminal reagent (Keygen, Nanjing, Jiangsu, China) and analyzed using Quantity One software (Version 4.4, BioRad, Hercules, CA, USA). Acetylation levels were normalized to total histone H3.

### 2.6 Real‐Time Reverse Transcription Polymerase Chain Reaction (RT‐PCR)

Total RNA was extracted from cardiac tissue using an RNA extraction kit (Takara, Kusatsu, Shiga, Japan). cDNA was synthesized from 1 μg RNA with a cDNA Synthesis Kit (Yeasen, Shanghai, China), followed by quantitative polymerase chain reaction (PCR) using SYBR Green kits (Yeasen, Shanghai, China). The primers' sequences of p300, CBP, *GATA4, Nkx2.5, MEF2C*, cTnI, α‐MHC, Acta1, and β‐actin were as follows: p300 (F) AGCCAAGCGGCCTAAACTC, (R) CGCCACCATTGGTTAGTCCC; CBP (F) GGCTTCTCCGCGAATGACAA, (R) GTTTGGACGCAGCATCTGGA; GATA4 (F) TGTGCCAACTGCCAGACTAC, (R) TGGGCTTCCGTTTTCTGGTT; Nkx2.5 (F) CCCAAGTGCTCTCCTGCTTT, (R) TTATCCGCCCGAGGGTCTTT; MEF2C (F) ATCCCGATGCAGACGATTCAG, (R) AACAGCACACAATCTTTGCCT; cTnI (F) CTCTGCCAACTACCGAGCCTA, (R) CTCTTCTGCCTCTCGTTCCAT; α‐MHC (F) GAAGAAAGTGCGCATGGACC, (R) GCCTGCTCGTCCTCAATTTTAC; Acta1 (F) TACCACCGGCATCGTGTTG, (R) GCGCACAATCTCACGTTCAG; and β‐actin (F) GATATCGCTGCGCTGGTCG, (R) CATTCCCACCATCACACCCT, respectively. The relative expression levels of target gene mRNA were evaluated using the 2^−^ *^ΔΔ^* ^Ct^ method, with β‐actin serving as the reference gene for normalization.

### 2.7 Chromatin Immunoprecipitation (ChIP) and Quantitive Polymerase Chain Reaction (Q‐PCR)

ChIP assays were carried out with a chromatin extraction kit and a ChIP kit (both from Abcam, Waltham, MA, USA) as per the manufacturer's guidelines. Immunoprecipitation employed antibodies against acetyl‐histone H3 (Lys4), acetyl‐histone H3 (Lys9), and acetyl‐histone H3 (Lys27). Precipitated DNA was quantified via quantitative reverse transcription polymerase chain reaction (qRT‐PCR) using a KAPA SYBR FAST qPCR kit (Kapa Biosystems, Cape Town, Cape Province, South Africa). Primer sequences targeting promoter regions of relevant genes were as follows: *Gata4* (F) CACTGACGCCGACTCCAAACTAA, (R) CGACTGGGGTCCAATCAAAAGG; *MEF2C* (F) CACGCATCTCACCGCTTGACG, (R) CACCAGTGCCTTTCTGCTTCTCC; and *Nkx2.5* (F) CTTCTGGCTTTCAATCCATCCTCA, (R) CGGGCAGTTCTGCGTCACCTA. Then the ChIP DNA Ct values were normalized to the input group (the total DNA collected after release) using the 2^−^ *^ΔΔ^* ^Ct^ method.

### 2.8 Statistical Analysis

Data are expressed as mean ± standard deviation (SD) and analyzed with SPSS 24.0 (IBM, Armonk, NY, USA). Comparisons among multiple groups were performed using one‐way ANOVA, and differences between two groups were assessed with a two‐tailed Student's *t*‐test. *p* < 0.05 was considered statistically significant.

## 3 Results

### 3.1 Curcumin Improved Manifestations of Mice With HF

After 35 days of continuous injection, mice in the HF group displayed signs including reduced appetite, lethargy, and unkempt fur. Following an additional 28 days of injection with either curcumin (Group D) or saline (Group C), the condition of the mice in Group C worsened, displaying increased lethargy, significantly decreased appetite, emaciation, and disheveled fur. In contrast, the mice in Group D showed improvement compared to Group C. Meanwhile, the mice in Groups A and B exhibited normal weight gain and displayed no abnormal symptoms.

BW, tail length, HW, and HW/BW ratio were measured (Table 1). No statistically significant differences in tail length were observed among the four groups. However, BWs in Groups C and D were significantly lower compared with Groups A and B (*p* < 0.01). Additionally, mice in Group C exhibited lower BW than those in Group D (*p* < 0.05). The HW/BW ratio was significantly elevated in Group C relative to the other three groups. Although Group D exhibited an elevated HW/BW ratio compared to Groups A and B, it remains somewhat smaller than that of Group C (*p* < 0.05). The results indicate that curcumin treatment improved the manifestation, BW, and HW/BW of HF mice.

**TABLE 1:** BW, TL, and HW/BW of mice in different groups.

Group		*N*	TL (cm)	BW (g)	HW/BW (ratio, %)
Control group	A	5	8.18 ± 0.15	27.14 ± 0.73	0.44 ± 0.02
	B	5	8.26 ± 0.19	26.04 ± 0.50	0.44 ± 0.02
HF group	C	5	8.20 ± 0.21	22.88 ± 1.15^*^	0.63 ± 0.12^*^
	D	5	8.18 ± 0.17	24.60 ± 0.71^#^	0.51 ± 0.02^#^

*Note:* Values are mean ± SD. *N*: the number of mice in each group.

Abbreviations: BW, body weight; HW, heart weight; TL, tail length.

^*^ *p* < 0.05 versus Group A.

^#^ *p* < 0.05 versus Group A and *p* < 0.05 versus Group C.

**FIGURE 1:**
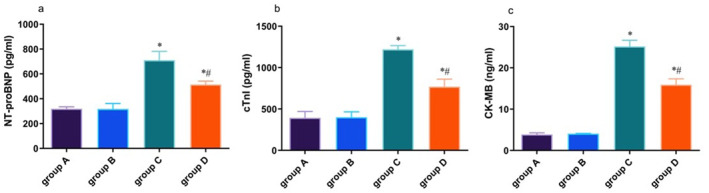
Levels of NT‐proBNP (a), cTnI (b), and CK‐MB (c) in different groups. * *p* < 0.05 versus Group A; # *p* < 0.05 versus Group C.

Serum levels of NT‐proBNP, cTnI, and CK‐MB were quantified using enzyme‐linked immunosorbent assay (ELISA). Results revealed markedly elevated serum levels of these biomarkers in the HF group relative to the control group (*p* < 0.05). Curcumin treatment in Group D led to substantial reduction in NT‐proBNP, cTnI, and CK‐MB levels compared to Group C (*p* < 0.05), suggesting that curcumin can reduce the level of biomarkers in mice with HF (Figure 1a–c).

### 3.2 Structure of Cardiomyocytes Improved After Treatment With Curcumin

**FIGURE 2:**
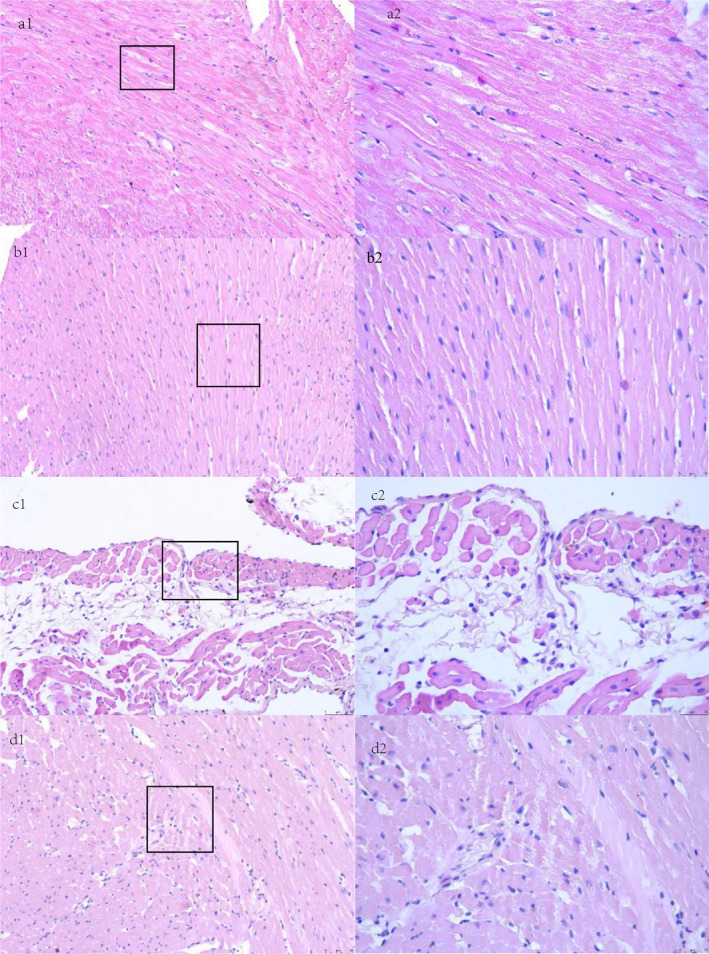
Structure of cardiomyocytes in different groups. The cardiomyocytes in Group C were broken, unarranged, and disordered, loosely and in a grid shape. In Group D, the cardiomyocytes were significantly better than Group C. A: Group A. B: Group B. C: Group C. D: Group D. a1, b1, c1, and d1: 200‐fold magnification; a2, b2, c2, and d2 show enlarged views of the areas marked by squares in Figures a1, b1, c1, and d1, respectively: 400‐fold magnification.

Cardiac tissue sections were stained with H&E and evaluated by light microscopy. Cardiomyocytes in the control groups appeared elongated and well‐organized, whereas those in the HF groups displayed structural disruption, disordered arrangement, loose organization, and a grid‐like pattern. Curcumin treatment (Group D) resulted in notable improvement in myocardial cell architecture relative to the untreated HF group (Group C; Figure 2a–c).

### 3.3 Expression of Cardiac Transcriptional Factors and HF‐Related Genes

**FIGURE 3:**
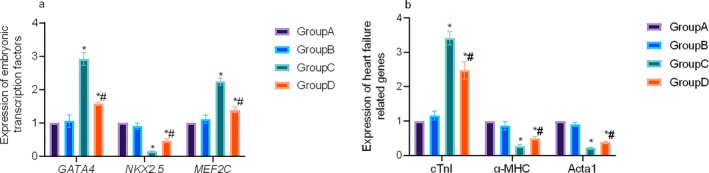
Expression of embryonic transcription factors (a) and HF related genes (b) in different groups. (a) Expression of *GATA4*, *Nkx2.5*, and *MEF2C*. (b) Expression of cTnI, α‐MHC, and Acta 1. * *p* < 0.05 versus Group A; # *p* < 0.05 versus Group C.

Expression levels of *GATA4*, *Nkx2.5*, and *MEF2C* in cardiac tissue were evaluated using RT‐PCR. Results revealed a significant upregulation of *GATA4* and *MEF2C* in the HF groups, accompanied by a marked downregulation of *Nkx2.5*. Curcumin intervention (Group D) led to reduced expression of *GATA4* and *MEF2C* and increased expression of *Nkx2.5*, with statistical significance (Figure 3a). These findings suggested that elevated *GATA4* and *MEF2C* expression might contribute to HF pathogenesis, whereas Nkx2.5 might exert a divergent role. Furthermore, expression of HF‐associated genes, namely cTnI, α‐MHC, and Acta1, was significantly increased in Group C, as detected by RT‐PCR. In Group D, their expression were lower compared to those in group C (*p* < 0.05, Figure 3b), indicating that inhibition of *GATA4* and *MEF2C* may inhibit or reverse the development of HF.

### 3.4 Acetylation of Histone H3 in Cardiac Tissues and the Promoter of *GATA4* and *MEF2C*

**FIGURE 4:**
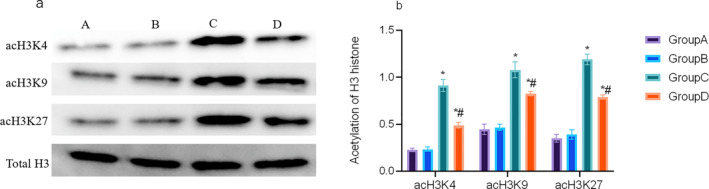
Western blot results of H3K4, H3K9, H3K27, and total histone H3 (a) and their quantitative analysis results (b). A: Group A. B: Group B. C: Group C. D: Group D.

Western blot analysis was performed to assess total histone H3, acetylated histone H3K4 (acH3K4), acetylated histone H3K9 (acH3K9), and acetylated histone H3K27 (acH3K27). Acetylation signals were normalized to total histone H3. Acetylation levels at each of these sites were significantly higher in the Group C relative to Groups A and B. Curcumin treatment (Group D) resulted in a pronounced reduction in H3K4, H3K9, and H3K27 acetylation relative to Group C (*p* < 0.05; Figure 4a,b).

**FIGURE 5:**
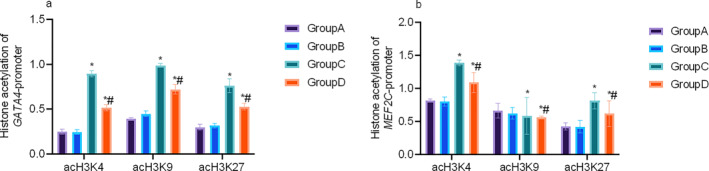
The acetylation of different sites in the promoter region of *GATA4* (a) and *MEF2C* (b). * *p* < 0.05 versus Group A. # *p* < 0.05 versus Group C.

The results above show that for the HF, the expression of embryonic transcriptional factors, *GATA4* and *MEF2C*, was reopened and may significantly influence the progression of HF. Building upon prior findings that histone acetylation influences embryonic transcription factors in cardiac development, we further examined its regulatory role in *GATA4* and *MEF2C* expression within the context of HF. Using ChIP‐qPCR with primers targeting the promoter regions of *GATA4* and *MEF2* *C*, we observed increased acetylation at H3K4, H3K9, and H3K27 in group C animals, effects that were significantly attenuated in group D (*p* < 0.05). The results indicated that acetylation at H3K4, H3K9, and H3K27 may contribute to the transcriptional reactivation of *GATA4* during cardiac pathogenesis, whereas in the region of *MEF2C*, the changes of acetylation at different sites were different. Within the *MEF2C* promoter region, acetylation patterns were site‐specific: Acetylation at H3K4 and H3K27 was elevated, whereas no significant change occurred at H3K9 (*p* < 0.05; Figure 5a,b).

## 4 Discussion

Heart failure pathogenesis involves a multifactorial process entailing mechanical stress and neurohormonal activation, ultimately promoting cardiomyocyte degeneration and loss [22, 23]. This process is marked by the reexpression of “fetal” heart genes and the downregulation of “adult” heart genes [24, 25]. The key to remobilizing gene expression is to change the structure of the chromatin, and epigenetics is an important mechanism to regulate chromatin structure [26, 27, 28]. Epigenetics is the process of changing the expression of genes through, for example, chromatin remodeling, DNA modification, histone modification, and micro‐RNA without changing the gene sequence, thus changing the phenotypes. Epigenetic modifications, particularly histone acetylation, are widely recognized as key regulators of gene expression during cardiac development. In eukaryotes, histone acetylation, mediated by histone acetyltransferases (HATs), represents a major epigenetic mechanism. This process involves the addition of acetyl groups to lysine ε‐amino groups on histone tails, neutralizing positive charge, reducing histone–DNA affinity, and facilitating transcriptional activation [29]. p300, a ubiquitously expressed acetyltransferase in cardiac tissue, was shown to be upregulated in both HF and cardiac hypertrophy. The inhibition of p300 can therefore improve cardiac hypertrophy and HF. It is suggested that epigenetic regulation of p300 expression can be used as a novel treatment strategy for HF.

Fetal genes including *GATA4*, *MEF2C*, *Tbx*, and *Nkx2.5* encode cardiac‐specific transcription factors. Their expression declines gradually during murine embryonic heart development and remains low and stable postnatally. These genes are crucial for cardiomyocyte differentiation; however, mutations or dysregulation can lead to cardiac malformations and congenital heart disease. Recent evidence also implicates their reexpression in the regulatory network of HF. Transgenic models demonstrate that postnatal cardiac overexpression or dominant‐negative inhibition of these genes can induce hypertrophy or HF. *GATA4*, an early expressed zinc finger transcription factor, is integral to heart formation [30]. Garry et al. found that *GATA4* was overexpressed in murine heart induced myocardial hypertrophy, indicating that postnatal reactivation of *GATA4* may drive maladaptive remodeling and contribute to HF progression [31]. Previous studies indicate that p300‐mediated acetylation enhances *GATA4* DNA‐binding activity and transactivation of hypertrophic response genes during HF [22]. *MEF2C*, another transcription factor, regulates pro‐hypertrophic gene expression and is inhibited by class II histone deacetylases (HDACs), thereby attenuating hypertrophy [32, 33]. Consistent with these reports, we observed elevated *GATA4* and *MEF2C* expression in mice with HF. Curcumin treatment suppressed their expression and ameliorated HF symptoms, suggesting that reexpression of *GATA4* and *MEF2C* promotes HF progression and that their inhibition may represent a therapeutic strategy.

However, the expression changes of *Nkx2.5* were different from those of *GATA4* and *MEF2Cc* in our study. In mice with HF, the expression of *Nkx2.5* was significantly reduced compared to the control groups, suggesting that *Nkx2.5* may not promote HF. Finding ways to promote the expression of *Nkx2.5* could possibly be beneficial for treating HF. Although it was reported that overexpression of *Nkx2.5* in transgenic adult mice could increase the expression of some HF related genes, such as ANF, BNP, and Myl2, no phenotype of myocardial hypertrophy or HF was observed [34]. In summary, the role of *Nkx2.5* in HF is still debated and needs more research.

Our results demonstrate the importance of fetal gene reexpression in the progression of HF. We demonstrate that acetylation of *GATA4*, *MEF2C  *promoter H3K4, H3K9, and H3K27 can promote the progression of HF, whereas inhibition of acetylation of these sites can alleviate or partially reverse HF. We found that histone acetylation of the fetal gene promoter is also site‐specific. The *GATA4* promoter region has more significant acetylation changes in H3K9, and the *MEF2C* promoter region has more significant acetylation changes in H3K27, with H3K9 acetylation remaining largely unaltered during HF. It is suggested that acetylation of *MEF2C* promoter H3K9 may not be involved in the regulation of HF. *Nkx2.5* is also an important transcription factor in heart development, but no significant reexpression occurred during HF, suggesting that Nkx2.5 may not be involved in the occurrence and development of HF. These findings imply that the reexpression of fetal genes mediated by epigenetic mechanisms in HF is selective and differs mechanistically from normal developmental regulation.

Nonetheless, HF is a highly complex clinical syndrome. Although aberrant activation of certain transcription factors is considered central to the progression of HF, other regulatory mechanisms—especially those involving noncoding RNAs—remain incompletely understood. Accumulating evidence demonstrates that microRNAs (miRNAs) can directly regulate target gene expression at post‐transcriptional and translational levels, as well as indirectly influence gene expression by interacting with other epigenetic mechanisms, including histone acetylation and histone methylation, thereby indirectly regulating downstream target gene expression [35, 36]. Investigating the epigenetic modifications during HF progression and elucidating their precise regulatory mechanisms still require further in‐depth research.

## 5 Conclusion

This study reveals that reactivation of fetal transcriptional factors (*GATA4* and *MEF2*C) contributes to the occurrence and progression of HF, with site‐specific acetylation modifications differentially regulating their expression. Importantly, inhibiting histone acetylation with curcumin can reduce the reactivation of fetal genes and ameliorate HF symptoms. Our results support the concept that epigenetic suppression of acetylation of fetal gene promoters could represent a promising therapeutic strategy and a preventive measure for HF. However, in addition to being a histone acetyltransferase inhibitor, curcumin may also exert anti‐inflammatory and antioxidant effects [37, 38, 39, 40]. However, whether the amelioration of HF symptoms following curcumin treatment in this model is solely attributable to histone acetylation inhibition requires further investigation. Moreover, there are no drugs specifically targeting *GATA4* or *MEF2C*. The potential off‐target effects of broad‐spectrum acetylation inhibitors on other transcription factors remain unclear. Therefore, developing agents that selectively inhibit *GATA4* and *MEF2C* transcription represents a promising therapeutic direction for HF.

## Author Contributions

Xinru Gan: experimental operations and data collection. Bo Pan: data analysis and manuscript writing. Lingjuan Liu: technical guidance for experiments. Mi Li: manuscript review. Huichao Sun: project management, funding provision, manuscript review and proofreading. All authors read and approved the final manuscript.

## Funding

The study was supported by Natural Science Foundation Projects of Chongqing Municipal Science and Technology Bureau (cstc2019jcyj‐msxmX0866).

## Ethics Statement

The study was approved and carried out according to the Guide for the Care and Usage of Laboratory Animals by the Animal Care and Use Committee of Children's Hospital of Chongqing Medical University (CHCMU‐IACUC20220629001).

## Conflicts of Interest

The authors declare no conflicts of interest.

## Data Availability

The data that support the findings of this study are available from the corresponding author upon reasonable request.
